# Bone-nerve crosstalk: a new state for neuralizing bone tissue engineering—A mini review

**DOI:** 10.3389/fmed.2024.1386683

**Published:** 2024-04-16

**Authors:** Laila A. Damiati, Marwa El Soury

**Affiliations:** ^1^Department of Biological Sciences, College of Science, University of Jeddah, Jeddah, Saudi Arabia; ^2^Department of Clinical and Biological Sciences, University of Torino, Torino, Italy; ^3^Neuroscience Institute Cavalieri Ottolenghi (NICO), University of Torino, Orbassano, Italy

**Keywords:** bone, peripheral nerve, tissue engineering, scaffolds, biofactors, growth factors

## Abstract

Neuro bone tissue engineering is a multidisciplinary field that combines both principles of neurobiology and bone tissue engineering to develop innovative strategies for repairing and regenerating injured bone tissues. Despite the fact that regeneration and development are considered two distinct biological processes, yet regeneration can be considered the reactivation of development in later life stages to restore missing tissues. It is noteworthy that the regeneration capabilities are distinct and vary from one organism to another (teleost fishes, hydra, humans), or even in the same organism can vary dependent on the injured tissue itself (Human central nervous system vs. peripheral nervous system). The skeletal tissue is highly innervated, peripheral nervous system plays a role in conveying the signals and connecting the central nervous system with the peripheral organs, moreover it has been shown that they play an important role in tissue regeneration. Their regeneration role is conveyed by the different cells' resident in it and in its endoneurium (fibroblasts, microphages, vasculature associated cells, and Schwann cells) these cells secrete various growth factors (NGF, BDNF, GDNF, NT-3, and bFGF) that contribute to the regenerative phenotype. The peripheral nervous system and central nervous system synchronize together in regulating bone homeostasis and regeneration through neurogenic factors and neural circuits. Receptors of important central nervous system peptides such as Serotonin, Leptin, Semaphorins, and BDNF are expressed in bone tissue playing a role in bone homeostasis, metabolism and regeneration. This review will highlight the crosstalk between peripheral nerves and bone in the developmental stages as well as in regeneration and different neuro-bone tissue engineering strategies for repairing severe bone injuries.

## 1 Introduction

Bone is a remarkable tissue that plays a vital role in the human body. It provides protection, support, and movement facility. Structurally bone consists of two main components: organic matrix (e.g., collagen), and inorganic minerals (e.g., calcium and phosphate) (1). There is a rich innervation of sensory and sympathetic nerve fibers in conjunction with the bone vascular system. Peripheral nerve fiber subtypes have a specific function within the bone (2). However, it has recently begun to be in the spotlight on the regulatory role of the bone and nervous system, which has been examined for many years (3–6).

Tissue engineering is a rapidly advancing field that holds great promise for treating various diseases and injuries (7). One area in which tissue engineering has shown significant potential is bone-nerve regeneration. Bone-nerve defects resulting from trauma, disease, or congenital abnormalities can be debilitating and difficult to treat using conventional methods. Due to that, with the advent of tissue engineering techniques, new possibilities have emerged for bone-nerve repair and regeneration. Crosstalk between bone and nerve systems has attracted the researcher's attention in different fields, such as clinical medicine, basic medicine, and biomaterials. While much progress has been made in developing biomaterials and scaffolds that mimic the structure of bone, one crucial aspect that cannot be overlooked is the role of nerves in this process (8, 9).

**Figure 1:**
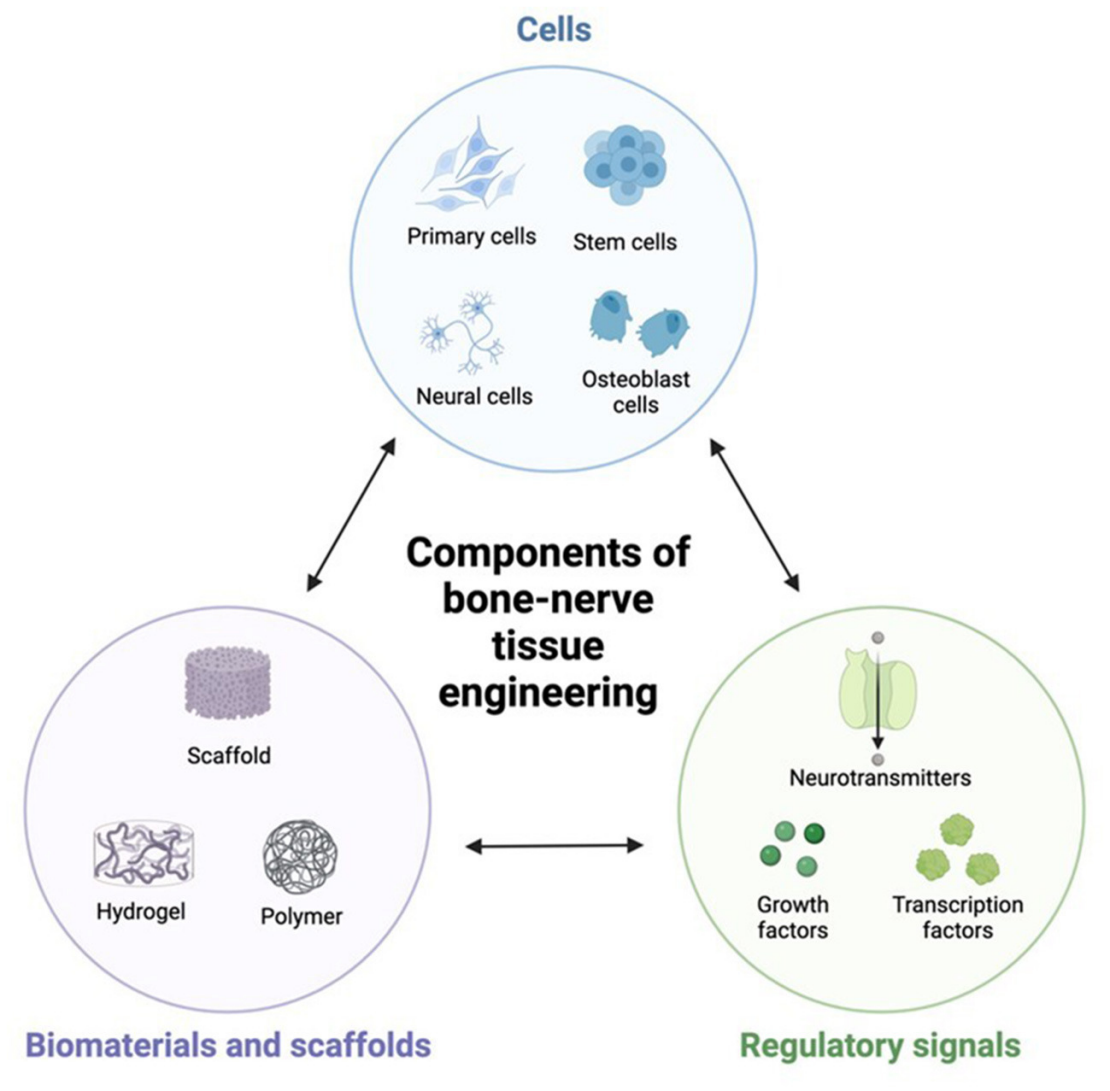
The three elements of bone-neuro tissue engineering.

Nerves play a vital role in bone regeneration by providing sensory input, controlling blood flow, and facilitating cells communication. They are responsible for transmitting signals that regulate cell proliferation, differentiation, and migration. Without proper innervation, tissue-engineered bones may lack functionality and fail to integrate with surrounding tissues (4, 10). In addition, nerves also contribute to pain perception during the healing process (11). Understanding how nerves interact with tissue-engineered constructs can help researchers develop new strategies to minimize pain and enhance patient comfort during recovery. To achieve successful nerve integration into tissue-engineered bones, scientists have explored various approaches such as incorporating nerve growth factors into scaffolds or utilizing stem cells capable of differentiating into both neuronal and osteogenic lineages. Therefore, this study summarizes the relationship between bone-nerve factors and functions, and their applications in bone-neuro tissue engineering (Figure 1).

## 2 Bone-nerve interaction in early developmental stages

Previously the function of the highly innervations in bones was just limited to pain perception, studies on denervated limbs that showed impaired bone development has proven the role of nervous system in bone development (12).

Structurally long bones are divided into three parts: the diaphysis, the metaphysis and the epiphysis, they are ensheathed by a thin connective tissue known as the periosteum, within which the majority of blood vessels and nerve fibers exist. Innervations are found in the bone marrow and the traverse nutrient canals in mineralized bone matrix as well (13).

Early studies of peripheral innervation to bones showed that nerve fibers start being visible at early embryonic stages, in the perichondrium of long bones, TrkA^+^ sensory fibers are visible at E14.5 in mouse embryo (14), while by E21 PGP9.5^+^ and GAP43^+^ nerve are visible in rat embryo (15). In the periosteum and bone marrow of the diaphysis CGRP^+^ sensory afferent fibers are visible at P3 (15). Autonomic fibers first appear in long bone postnatally. Sympathetic NPY^+^ nerve fibers are first visible in the bone marrow of the mouse femur and tibia at P6. Both NPY^+^ and TH^+^ fibers are evident in perichondrium of the epiphysis and the cartilage canals at P10 (12, 14).

The nervous system is involved in bone growth and limb development where sensory and autonomic innervation play a crucial role in bone formation, a study by Tomlinson has shown that NGF expression in the developing endochondral bone coincides with vascularization and osteochondral progenitor cell expansion. Inhibition of TrkA signaling or deletion of NGF in perichondral osteochondral precursor cells over this time frame disrupts ossification of the primary and secondary ossification centers and impairs postnatal bone mass and length, thus proofing that NGF is essential for bone progenitor cell differentiation and mineralization (16). Another study showed that the sensory neuropeptide calcitonin gene-related peptide (CGRP) act at earlier stages in osteoclast development and inhibits osteoclastic bone resorption and enhances osteoblastic bone formation (17). Neuropeptide Y (NPY) is involved in the regulations of bone metabolism and homeostasis, it can stimulate proliferation, migration, promote osteoblastic differentiation and play crucial roles in the bone development (18). Indeed, in a study with global NPY deletion male mice had shown a smaller femoral cortical cross-sectional area (−12%) and reduced bone strength (−18%) while female mice had shown no alterations in bone mass, suggesting a sex-specific effect (19).

A study by Kong et al. uncovered a novel brain-skeleton axis involving NSCs in the mammalian brain, demonstrating the role of bone regeneration through perilipin 5 (PLIN5)-driven lipid metabolism modulation. This study found that the homocysteine (Hcy) negatively affected the osteogenic and angiogenic processes, while the NSC-Exo restored these impairments. Furthermore, the mecobalamin (a neurotrophic drug) enhanced the protective effects of NSC-Exo by increasing the PLIN5 expression. On the other hand, mechanistically, the NSC-derived PLIN5 reversed Hcy-induced lipid metabolism imbalance and aberrant lipid droplet accumulation through lipophagy-dependent intracellular lipolysis. This effects of PLIN5-deriven endocrine play a role on new bone formation and vascular reconstruction in models with elevated homocysteine and high-fat diet through intracerebroventricular administration of mecobalamin and/or AAV-shPlin5 (20).

Skeletal interoception refers to the perception and interpretation of internal signals that are arising from skeletal system. This sensory mechanism involves the mechanical changes, biochemical signals, and proprioceptive information within bones and joints (21, 22). However, it was known that the interoception was associated with visceral process, and recent research has showed the importance of skeletal interoception in understanding the dynamic interactions between bones and nervous system (23).

## 3 Bone-nerve and its role in tissue engineering of bone regeneration

Bone regeneration is a complex process involving different molecular mechanisms with numerous complicated interactions through stem cell regulation, cytokines, peptide, hormones, and environmental factors (4, 24, 25). Among these many regulatory factors, the neural regulation of bone regeneration is attracting many interests. Previous studies have been described the rich nerve network that distributed in all types of bone. The nervous system regulates bone regeneration by targeting two basic functional cell types, osteoblast, and osteoblasts. These cells are responsible in skeletal model changes, bone growth and remodeling (26). The nerve fibers that are spread ion the bone can transmit nerve signals to muscle and tendon tissue which can influence the compression or stretching of the bone through different factors such as mechanical environment (27). To achieve successful nerve growth towered regenerated bones, cells, scaffolds, and biofactors with specific properties are required.

### 3.1 Cells

The interactions process between nerve and bone cells involves series of cellular properties that work in harmony to ensure successful bone-nerve regeneration. These properties include cell adhesion, cell signaling, and cell migration. Cell adhesion allows cells to stick together and form cohesive structure. In the context of nerve growth toward regenerated bones, cell adhesion plays a vital role in guiding nerves along the desired path. By adhering to each other and forming bridges, cells create a scaffold for nerve fibers to grow upon (28). In cell signaling during nerve regeneration, specific signaling molecules are released at the site of bone regeneration. These molecules act as guidance for growing nerves directing them toward the regenerating bone tissue (10, 29). In addition, certain type of cells migrates toward the regenerating bone tissue and align themselves along its surface. This alignment provides physical guidance for growing nerves and helps them navigate their way toward the desired destination (28, 30).

Stem cell, especially mesenchymal stem cell (MSC) can be isolated from a variety of tissues such as bone marrow, adipose tissue, and the synovial fluid. MSC has a unique feature due to its ability to differentiate into different cell types such as osteoblast and chondrocyte (31).

### 3.2 Scaffolds

Scaffolds with specific structures can provide support and guidance for cell growth and tissue regeneration. They can be made from various materials, such as metals, polymers, or ceramics, which possess specific properties to promote bone-nerve growth (7, 32). For instance, the scaffold's surface topography can be engineered to mimic the natural extracellular matrix, providing cues for bone-nerve cells to align and extend along desired pathways. There are specific requirements the scaffolds need to meet for successful bone-nerve regeneration: (1) Good biocompatibility that facilities the bone and nerve adhesion and growth, (2) suitable mechanical properties where the scaffold should have adequate stiffness to withstand physiological loads and prevent collapse while allowing for cellular infiltration and nutrient diffusion, (3) appropriate elasticity to mimic the surrounding tissues' mechanical properties and facilitating proper integration with the host bone, (4) good bone or nerve conductivity and inductivity, which help bone or nerve to deposit on the material surface and helps recruit stem cells to induce the osteogenic and neurogenic differentiation, and (5) control the release of bioactive factors (32–34).

Various types of scaffolds have been developed with different properties and advantages. One commonly used of scaffold is the natural scaffolds that is derived from biological sources such as collagen or decellularized extracellular matrix (ECM). These types of scaffolds closely mimic the natural composition, which making them highly compatible. However, the mechanical properties of natural scaffolds may not be optimal foe load-bearing applications. On the other hand, the synthetic scaffolds are fabricated using synthetic materials such as metals, ceramic, or polymers. These materials can be engineered to possess specific mechanical properties, that allowing for better control over their degradation rate and strength. However, they may lack the necessary bioactivity for promoting cell adhesion and differentiation (7, 35, 36).

Hybrid scaffold is a combination of both natural and synthetic materials to harness the advantages of both types. By reinforcing natural scaffolds with synthetic materials, or incorporating bioactive components into synthetic scaffold, the hybrid scaffolds can provide an optimal microenvironment for tissue engineering (36, 37).

### 3.3 Biofactors

Biofactors such as growth factors play a crucial role in guiding nerve growth toward regenerated bones. Nerve regeneration is a complex process that requires the coordination of various cellular and molecular events (38). The interaction between nerves and bones is essential for proper bone healing and regeneration. During nerve regeneration, growth factors attract nerve cells toward the site of injury or bone defect. This migration is essential for establishing connections between nerves and bones, allowing for good communication and function (39). Furthermore, growth factors stimulate the proliferation of nerve cells. By promoting cell division, these factors increase the number of nerve cells available for regeneration which enhances the chances of successful nerve regrowth toward the regenerated bones (10). In addition, growth factors also have a role in promoting angiogenesis, which is the formation of new blood vessels. Blood vessels are essential for delivering oxygen and nutrients to regenerating tissues, including nerves and bones. By stimulating angiogenesis, growth factors ensure an adequate blood supply to support nerve growth toward regenerated bones (40, 41). Moreover, growth factors can modulate the expression of specific genes involved in nerve regeneration processes. They can upregulating genes responsible for axonal guidance and outgrowth while downregulating genes that inhibit nerve regrowth (42).

## 4 Growth factors for bone regeneration

One of the most important factors for bone regeneration is transforming growth factor beta (TGF-β). TGF-β is able to stimulate the MSCs differentiation into osteoblast that are responsible for bone formation. It also plays a role on stimulating the ECM proteins production that are necessary for bone repair Transforming growth factor-β in stem cells and tissue homeostasis (43). Another significant growth factors are bone morphogenic proteins (BMPs), which are a group of GFs that induce osteogenesis and stimulate bone formation. BMPs play a crucial role in initiating the early stages of bone repair by attracting MSCs to differentiate into osteoblasts (44). Insulin-like growth factor (IGF) is another important GF that enhances cell proliferation and differentiation. It stimulates osteoblast activity which leads to increase bone formation during bone healing process (45). Vascular endothelial growth factor (VEGF) is essential for angiogenesis, the new blood vessels formation. Adequate blood supply is essential for delivering oxygen and nutrients to the site of injury to promote bone healing (46) (Table 1).

**Table 1:** List of bone- and neuro- growth factors.

**GF**	**Function**	**References**
*TGF-ß1*	Osteogenic and chondrogenic differentiation	(47–49)
*BMP2*	Osteogenic differentiation	(47, 50–53)
*BMP7*	Osteogenic differentiation and regulates proliferation of neural progenitor cells	(51, 54, 55)
*HGF*	Cell proliferation, morphogenesis, and angiogenesis	(47, 56)
*VEGF*	Angiogenesis	(47, 57, 58)
*CTGF*	Angiogenesis and ECM remodeling	(47, 58)
*FGFs*	Angiogenesis, proliferation, and osteogenic differentiation	(47, 59)
*PDGFs*	Cells proliferation, ECM synthesis, and angiogenesis	(47, 60)
*NGF*	Osteoblast differentiation markers induction	(61)
*BDNF*	Vascularization, Osteogenesis	(62, 63)
*NE*	Osteoblast proliferation, Osteogenesis	(64, 65)
*CGRP*	Osteogenesis	(66, 67)
*SP*	Osteogenesis (low concentration), Proliferation (high concentration)	(68)
*Ach*	Osteoblasts proliferation and differentiation	(69)
*NPY*	Osteoblast lineage differentiation, bone homeostasis	(70, 71)

## 5 Growth factors for nerve regeneration

Nerve regeneration is a complex process in which several morphological and molecular changes occurs in the proximal and distal nerve stumps, in response to nerve injury various neurotrophins, neurotransmitters and neuropeptides are released that synergize together and play different roles in nerve and bone regeneration (Table 1), they exert their action by modulating several pathways including Wnt, Erk1/2 and AKT pathways Nerve Flex modulation by exercise training and mechanical loading positively affect nerve and bone regeneration by increasing the expression of NGF and Wnt signaling activation (72).

### 5.1 Neurotrophins

#### 5.1.1 Nerve growth factor

Nerve Growth Factor (NGF) is a member of neurotrophins family playing an important role in growth and survival, other members are brain derived neurotrophic factor (BDNF), neurotrophin-3 (NT-3), and neurotrophin 4/5 (NT-4/5). NGF was first discovered by Rita Levi-Montalcini in the 1950s, and this work eventually led to the Nobel Prize in Physiology or Medicine in 1986 (73). NGF biological action is mediated by binding and activating receptor tyrosine kinase trkA or a transmembrane glycoprotein pan-neurotrophin receptor p75NTR (74). Although these factors were discovered because of their actions during development, where a study by Smeyne et al. has demonstrated that mice embryos with homozygous deletion of the Trk gene, suffer from neuronal cell loss in trigeminal, sympathetic, and dorsal root ganglia (75), now they are known as well for their roles in regeneration. NGF has demonstrated to play an important role in nerve regeneration. NGF has low expression levels in healthy nerves, it is upregulated in response to peripheral nerve injury which proves its role in nerve regeneration (61), indeed several studies have been carried out on animal models where exogenous NGF administration had shown promoted peripheral nerve growth and reestablishment of the functional activities of peripheral nerve fibers and damaged neurons. Repairing a transected nerve with silicone chambers filled with 1 mg/ml NGF resulted in an increased number of myelinated axons and 58% increased myelin sheath thickness compared to the control saline group (76). In another study shows that NGF is crucial to nerve regeneration by activating autophagy in Schwann cells and accelerating myelin debris clearance, a crucial prerequisite for a successful Wallerian degeneration and subsequent nerve regeneration, thus NGF promote axon and myelin regeneration at early stage of PNI (77). It has been shown that NGF plays a role in bone development as well, where NGF-TrkA signaling in sensory nerves is essential for early innervation, normal vascularization, and formation of both primary and secondary ossification centers of endochondral bone during late embryogenesis thus maintaining a normal femoral length and volume (16). While most studies are focused on NGF exerting its bone regenerating role via TrkA receptor, another study have shed the light on the NGF role binding to its low affinity receptor p75, in a cranial bone injury model it was identified the role of p75 signaling pathway in coordinating and stimulating skeletal cell migration during early bone repair where mice lacking NGF in myeloid cells or p75 in osteoblasts demonstrated reduced migration of osteogenic precursors to the injury site with consequently delayed bone healing (78). The influx of NGF expressing macrophages following cranial bone injury play a role in bone repair by inducing skeletal sensory nerve ingrowth that positively regulate bone repair, Genetic disrupt in NGF expressing macrophages or locoregional deletion of NGF delayed reinnervation and blunted the repair process, similarly delayed reinnervation and repair was induced by the inhibition of TrkA catalytic activity (79). NGF can be therapeutically used in accelerating bone healing in a tibial fracture model where a local injection of β-NGF during the endochondral/cartilaginous phase promoted cartilage to bone conversion (62). Tissue engineered NGF carrying hydrogels have shown a positive effect on promoting bone regeneration. In a rabbit mandibular distraction osteogenesis model, the enrichment of collagen/nano-hydroxyapatite/alginate hydrogel with NGF allowed its delayed release, prevention from inactivation and a prolonged *in vivo* NGF concentration, the group receiving the NGF enriched hydrogel has shown a superior bone histology where new bone formation was observed compared to saline control group (63). These results were in line with a previous study that has used a hydrogel of collagen/hydroxyapatite as a NGF delivery system in a model of rat calvarial defect, the treated group has demonstrated an increase in bone ingrowth and mass compared to control group (64). Due to NGF poor pharmacokinetic properties another study tested a highly selective TrkA agonist, Gambogic amide (GA), the results show that GA can improve bone repair by increasing the mRNA expression of osteoblastic and osteolytic differentiation markers, thus proofing that GA, like NGF, may promote fracture healing through promoting both osteoprogenitor differentiation and mineralization (65).

#### 5.1.2 Brain derived neurotrophic factor

Brain-derived neurotrophic factor (BDNF) is neurotrophin family, it is known to exert its action by binding to one of its two receptors, tropomyosin receptor kinase B (TrkB) or the common neurotrophin receptor, p75NTR. BDNF has been extensively studied for its roles in neural development, BDNF ko mice are embryonically lethal, proofing that its essential during developmental stages (80). BDNF play a wide role in the central nervous system mainly in long term potentiation (LTP) thus enhancing the synaptic plasticity, learning and memory (80). In the peripheral nervous system it was shown that BDNF plays a role in peripheral nerve regeneration as it stimulates the actin deposition and polymerization in the regenerating nerve growth cone (81). It was found that BDNF expression is upregulated in response to peripheral nerve injury, in a model of sciatic nerve lesion which was deprived from BDNF by antibody treatment resulted in a dramatic reduction both in the number of myelinated axons distal to the lesion and the elongation of regenerating axons which led to a significant retardation of nerve regeneration and remyelination of injured sciatic nerves. Thus, we conclude that endogenous BDNF is required for regeneration and remyelination of the peripheral nerve after injury (82). BDNF has shown to play a role in bone fracture healing, in a study that analyzed the expression pattern of BDNF and its receptor from fracture gaps samples obtained from during different phases of bone healing. BDNF and TrkB expression were determined in early fracture hematomas expressed by hematopoietic cells, and in later stages of fracture healing by active osteoblasts, while its expression is totally absent in healthy mature bone tissue (66). Its action can be mediated indirectly by improving vascularization and innervation of the injured bone, as was shown in an *in vivo* study using a tissue engineered Tricalcium phosphate scaffold enriched with BDNF enhanced the multipotency of the seeded Bone mesenchymal stem cells (BMSCs) leading to the generation of a better innervated and vascularized bone tissue, thus promoting neurogenesis and osteogenesis (83). *In vitro* studies stimulating MC3T3-E1 cells (osteoblast lineage cell line) with BDNF has shown to induce osteogenesis by upregulating the expression of osteoblast differentiation marker osteocalcin (84).

Bone-derived modulators are showing potential roles in neurological disorders. For instance, Osteopontin (OPN) is highlighted with dual roles, suggesting that low levels may inhibit wound healing, while high levels may induce excessive tissue injury in Parkinson's disease (67, 85). Furthermore, an unexpected finding indicates that bone-derived Osteocalcin (OCN) is crucial for young blood for rejuvenation as it may contain molecules with rejuvenating effects on the brain (86). Lipocalin-2 (LCN2), another bone-derived hormone, was found to play a role in activating the anorexigenic pathway by binding to the melanocortin-4 receptor of the hypothalamus after crossing the blood-brain barriers (BBB) (87). Furthermore, previous studies have emphasized that bone marrow-derived mesenchymal stem cells (BMMSCs) play a significant role in various neurological disease treatments, such as Alzheimer's and ischemic (88). A review paper by Chen et al. provided a comprehensive overview of the functions of bone-derived modulators, including OCN, OPN, LCN2, BMMSCs, hematopoietic stem cells, and microglia-like cells and their implications on the pathogenesis of neurological disorders (85, 89, 90). However, the roles of these modulators in the brain still need to be completed, which requires further investigations to understand their full therapeutic potential to develop a particular tool for neurological disorders.

### 5.2 Neurotransmitters and neuropeptides

#### 5.2.1 Norepinephrine

Norepinephrine is a member of catecholamine family of tyrosine-derived neurotransmitters in the sympathetic nervous system exerting their action by binding the adrenergic receptor (AR) alpha and beta isoforms, which are present on both osteoblasts and osteoclasts (68). α-AR signaling in osteoblastic cells can upregulate proteins that are related to osteogenesis, thus promoting bone formation (91). NE plays a role in chondrocyte metabolism, in an *in vitro* study where Primary costal chondrocytes were isolated and stimulated with NE, it was found that the apoptosis rate of chondrocytes was decreased, and NE can regulate the proliferation of osteoblasts, osteoblast-like cells, and mesenchymal stem cell lines (68). β-AR are expressed in osteoblasts and osteoclasts it is mainly involved in bone metabolism regulation (92).

#### 5.2.2 Calcitonin gene related peptide

Calcitonin gene related peptide is a members of the calcitonin family, mainly it is a nociceptive neurotransmitter acting on one of two receptors: calcitonin gene-related peptide receptor (CLR) and receptor activity modifying protein 1 (RAMP1) (93). CGRP plays a crucial role in bone fracture healing, in a study that collected plasma from patients with fracture neck of femur it was found to be highly elevated after 24 h compared to healthy controls (69). In an *in vivo* study on CGRP-deficient mice following femoral osteotomy, CGPR was found to be elevated in WT mice serum, CGRP deficient mice showed highly impaired bone regeneration and a reduction in the number of osteoblasts, genome wide expression analysis has shown that CGRP induces the expression of specific ossification linked genes (94). These results were in line with a previous study performed on selectively abolished CGRP production mouse model, CGRP-deficient mice displayed a low bone mass phenotype, reduced bone formation rate and developing osteopenia (95). In contrast targeted overexpression of CGRP in mice increased bone formation rate which resulted in an increased bone volume and density (96). CGRP stimulates osteogenesis and upregulate different osteoporotic differentiation-related genes, CGRP *in vitro* stimulation of bone marrow-derived mesenchymal stem cells (BMSCs) extracted from an osteoporotic female rats induced proliferation at 7 days cultures, at longer cultures more than 14 days it promoted BMSCs differentiation into osteoblasts that formed calcified nodules after long-term culture (70). CGRP maintain the same osteogenic differentiation capacity in BMSCs derived from aged rats (71). CGRP also protects bones by inhibiting the osteoclastogenesis a process of bone breaking down (97). CGRP as well acts as a bone metabolism modulator through osteoblast and osteoclast associated mechanisms (98). Local CGRP administration in a distraction osteogenesis model have demonstrated enhanced angiogenesis, increased endothelial progenitor cells (EPCs) population and promoted blood vessel formation and bone regeneration (99).

#### 5.2.3 Substance P

Substance P (SP) is a member of the mammalian tachykinin family, mainly present in sensory nervous system playing an important role in numerous biological processes related to pain transmission. SP is often co-released with CGRP and it mediates its biological effects through binding to neurokinin-receptor 1 (NK-R1) also known as tachykinin 1 receptor (TACR1) (100, 101). SP positive nerve fibers are widely distributed in various bone tissues, especially in the metabolically active parts, such as the periosteum. Similar to CGRP, SP as well plays a crucial role in bone fracture healing, with highly elevated levels in fractured bone patients (69). Nk-R1 is expressed by both osteoblasts and osteoclast precursors, the balance between osteoclastic bone resorption and osteogenic bone formation is the key factor in maintaining normal bone mass (101). Indeed, in an *in vitro* study the concentration-dependent effects of SP Signaling on primary osteoblast and osteoclast progenitor cells throughout the period of cell differentiation was investigated, SP stimulates BMSC proliferation and mineralization at higher concentrations, while lower SP concentrations increase BMSC osteogenesis at later stages of osteoblastic differentiation. (102).

#### 5.2.4 Acetylcholine

Acetylcholine (Ach) is the first neurotransmitter to be discovered, an essential mediator in central and peripheral nervous system. It is synthesized by choline acetyltransferase (ChAT) enzyme and degraded by Acetylcholinesterase (AChE), and acts by binding to two types of receptors ACh acts on two main types of receptors: nicotinic and muscarinic acetylcholine receptors. The cholinergic system is widely distributed in many non-neural tissues as well (103). Ach receptors and the Acetylcholine enzymes (ChAT and AChE) were found to be expressed in both osteoblasts (104) and osteoclasts (105). The expression level of AChE changes during embryonic and postnatal bone developmental stages suggesting that it is involved in bone development and remodeling (103).

Ach could play a role in osteoblasts proliferation and differentiation (106).

Ach plays a gender specific role in bone remodeling following peak bone mass acquisition, in a study on choline transporter (ChT) heterozygous mice it was found that a reduced central Ach synthesis leads to a decrease in bone mass just in young female mice. This effect was rescued by galantamine a blood brain barrier permeable acetylcholinesterase inhibitor (AChEI) that caused an increase in brain Ach levels (107). The gender specific effect of Ach was demonstrated in another study concerning bone mechano-adaptation, osteocyte targeted conditional knockout mice with a deletion in the cholinergic receptor subunit α1 showed sexually dimorphic differences in bone formation rates and bone structure between experimental group and control, reductions in female bone geometry could be rescued by anabolic loading, not in male (108).

#### 5.2.5 Neuropeptide Y

Neuropeptide Y (NPY) is a highly conserved neuropeptide that is important for various physiological processes expressed in both nerve system and bone tissue, it acts by binding to its receptors Y1, Y2, Y4, Y5, and Y6, Y1 and Y2 are mainly expressed in osteoblasts, osteocytes and bones tissues (109). They play a major role in osteoblast lineage differentiation (110) as well as bone homeostasis, during aging it is secreted by osteocytes, they differentiate BMSCs into adipogenic cells, NPY deletion form osteocytes results in an increase in the bone mass (111).

Contrary NPY have a positive effect on bone fracture healing, in response to a femoral injury it was found that the transcriptional and protein levels of NPY and its receptors are upregulated during bone repair (112), while the use of NPY inhibitors inhibit fracture healing (113). It was also demonstrated that NPY promotes neuroprotection and NPY- deficient mice show an impaired regeneration (114).

Table 2 summarizes some examples of the impact of cells, scaffolds, and biofactors on bone-nerve regenerations.

**Table 2:** Examples of cells, scaffolds, biofactors on bone-nerve tissue engineering.

**Cell type**	**Scaffold**	**Biofactor**	**Findings**	**References**
Human bone marrow mesenchymal stem cells (hBMMSCs)	poly(ethylene glycol)-b-poly(lactic-co-glycolic acid)-b-poly(N-isopropylacrylamide) (PEG–PLGA–PNIPAM) hydrogel	Aspirin (ASP) and miRNA-222	ASP and miRNA-222 has an ability to induce hBMMSCs differentiation into neural-like cells through Wnt/β-catenin/Nemo-like kinase signaling.	(115)
Osteoblasts	Porous biphasic calcium phosphates (BCP)	Nerve growth factor (NGF)	The effect of neuro-osteological interactions through combinatorial treatment with NGF and BCP has an ability to promote osteogenesis and bone formation.	(116)
*In vivo:* defects on the parietal bone of the skull	Collagen	β-nerve growth factor (β-NGF)	β-NGF promoted neurogenesis and may modulate angiogenesis by promoting nerve regeneration in collagen bone substitute-filled defects.	(117)
Osteoblasts	Collagen	Norepinephrine (NE) and Ca^2+^	The scaffolds with the presence of NE and Ca^2+^ showed good mechanical and biological properties for promoting bone regeneration.	(118)
Dental germ	Nanofibrous polycaprolactone	Nerve growth factor (NGF)	These combinations allow a complete functionality and tissue homeostasis of the tooth.	(119)
*In vivo:* type 1 diabetes mellitus (T1DM) mice with tibial bone defects	PLGA and titanium (Ti)	Melatonin	PLGA@MT-Ti showed dual effects through activating the BMP-4/WNT pathway and attenuating ROS overproduction to promote osteogenesis and osteointegration at the Ti-bone interface.	(120)
BMSCs	Ti	Semaphorin 3A (Sema3A)	The Ti surfaces with the presence of Sema3A illustrated an effect on bone remodeling markers and downstream modulation of osteoclastic activity.	(121)
BMSCs	β-tricalcium phosphate (α-TCP)	Sensory nerve/BMSCs	The sensory nerve fibers could grow into the pores of the bone graft rapidly, and increase the expression of calcitonin gene-related peptide which help in the regulation of bone formation and blood flow.	(122)
BMSCs, umbilical vein endothelial cells, neural stem cells	Silk-hydroxyapatite (HA)	BMP-2, VEGF and NGF	Due to upregulation of osteoblastic genes expression (Runt-related transcription factor-2, Osteopontin, Bone Sialoprotein), the growth factors showed a synergistic affect in terms of osteoblastic differentiation.	(123)
iPSC-MSCs	HA scaffolds	Sema3A	Modifying cells in the presence of Sema3A represent a promising strategy to optimize tissue engineering -based strategy in bone repair.	(124)
BMMSCs, endothelial progenitor cells	Silicon	Sema3A, Sema4D	Sema3A production by sensory nerves on silicon surfaces stimulates the osteogenesis and angiogenesis functions.	(125)
BMMSCs, adipose mesenchymal stem cells (ASCs)	PLGA	Sema3A	Sema3A increased the expression of multiple Wnt genes that are responsible in osteogenic differentiation.	(126)
hBMSCs	α-TCP	Brain-derived neurotrophic factor (BDNF)	BDNF promotes hBMSC osteogenesis and neurogenesis and may indirectly promote osteogenesis through increased neurogenesis.	(83)
*In vivo:* osteoporotic muscarinic acetylcholine receptor M3 (M3 mAChR) knockout (KO) mice	α-TCP-HA	BDNF	BDNF-functionalized scaffolds promoted fracture healing in non-osteoporotic bone.	(127)
BMSCs	Hydrogel	EGFL-like 1 (*Nell1*)	Hydrogel with Nell1-modified-EVs showed a potential role for induced osteoblast lineage commitment program of BMSCs and bone repair.	(128)
BMSCs	Laponite	NGF	This combination has an ability to improve regeneration of vascularized bone tissue by releasing gene-related peptide from sensory nerves.	(129)
NSCs and BMSCs	Hydrogel with magnesium-ion-modified black phosphorus	-		(130)

## 6 Conclusions

Nerve and bone injuries are common in the modern days, both are limiting factors that negatively affect the quality of patient's life and is considered an economical burden on the society. While peripheral nervous system can retain a limited regeneration capacity based on the severity of the injury. Bone fractures usually need surgical intervention. Our bodies are complicated systems which in need of a proper communication between its different systems, the functioning of one system is dependent on the proper functioning of another. The nervous and skeletal system is a perfect example demonstrating this interaction and functional cooperation. Cross-talks between the nervous and the skeletal system ensure metabolic and functional homeostasis. Developmental signals from the nervous system ensure a healthy functioning skeletal system. This interaction makes neurotrophins, neurotransmitters and neuropeptide enrichment of bone scaffolds a promising strategy for severe bone fractures repair.

## Author contributions

LD: Writing – review & editing. ME: Writing – review & editing.
